# Comparative analysis of adolescent intramedullary nailing and locking plate fixation for femoral shaft fractures

**DOI:** 10.3389/fsurg.2025.1614146

**Published:** 2025-07-17

**Authors:** Jian Huang, Yiqun Bian

**Affiliations:** Department of Orthopedic, Liaocheng People’s Hospital, Liaocheng, Shandong, China

**Keywords:** adolescent femoral shaft fractures, antegrade intramedullary nailing, bone healing, inflammation, lateral plate fixation

## Abstract

**Background:**

Femoral shaft fractures in adolescents are commonly treated with either antegrade intramedullary nailing (AIN) or lateral plate (LP) fixation. This study compared the clinical outcomes of these two methods.

**Methods:**

Adolescent patients with femoral shaft fractures were randomly assigned to either the AIN group (*n* = 62) or the LP group (*n* = 62). Surgical parameters, early recovery metrics, levels of inflammatory cytokines, pain mediators, and bone metabolism markers were assessed.

**Results:**

The AIN group had significantly shorter incision lengths (6.8 vs. 8.5 cm, *p* < 0.001) and lower intraoperative blood loss (120 vs. 170 ml, *p* < 0.001) compared to the LP group. AIN patients began weight-bearing activities earlier (34 vs. 47 days, *p* < 0.01). Three months post-operation, the AIN group showed superior proximal femoral geometry and hip function, with a higher non-arthritic hip score (NAHS, 87 vs. 75, *p* < 0.001). One-week post-operation, the AIN group had lower serum levels of inflammatory cytokines and pain mediators, indicating a reduced inflammatory response and less postoperative pain.

**Conclusions:**

AIN offers significant advantages over LP in treating adolescent femoral shaft fractures, including reduced surgical trauma, faster early recovery, lower inflammatory response, less postoperative pain, and enhanced bone healing.

## Introduction

Femoral shaft fractures in adolescents, while relatively uncommon, represent a significant clinical challenge. In our department, these fractures accounted for 1.98% of all pediatric fractures (124 cases out of 6,268 total pediatric fractures). Despite their rarity, these injuries demand prompt and effective surgical intervention to ensure optimal healing and functional recovery (1, 2). These fractures often resulting from high-energy trauma such as sports injuries or vehicular accidents, present unique challenges due to the ongoing growth and development of the adolescent skeletal system. The primary goals in managing these fractures are to ensure proper healing, restore full function, and prevent any long-term complications that could affect the mobility and quality of patient life (3, 4). Given the complexity and potential impact of these fractures, selecting the most effective surgical treatment is critical for optimizing outcomes.

Treating displaced femoral shaft fractures typically requires surgical intervention. The choice of treatment method is influenced by factors such as the age, weight, fracture type, and socioeconomic status (5, 6). Two prevalent surgical techniques for treating adolescent femoral shaft fractures are antegrade intramedullary nailing (AIN) and locking plate (LP) fixation (3, 6, 7). AIN involves inserting a metal rod into the marrow canal of the femur, providing internal support that allows for early weight-bearing and mobilization (8). This procedure involves inserting a metal rod (nail) into the medullary canal of the bone from the proximal end, typically through the hip for femoral fractures or the knee for tibial fractures. The nail is then secured with screws at both ends to provide stabilization and promote healing. AIN offers several advantages, including minimal disruption to the fracture site, preservation of the periosteal blood supply, and early weight-bearing for patients (9, 10). It is particularly effective for diaphyseal fractures, where it provides strong internal support and allows for relatively quick recovery. LP fixation is another common method for treating fractures, especially in cases where the bone is highly comminuted or where there is a need for angular stability (11). This technique involves the application of a plate along the bone with screws that lock into the plate, creating a fixed-angle construct. Unlike traditional plating methods, locking plates do not rely on bone compression for stability, making them suitable for osteoporotic bones and fractures near joints. LP fixation provides robust stabilization, maintains the alignment of the fracture, and minimizes the risk of secondary displacement. It is often used in complex periarticular fractures and where intramedullary nailing is not feasible.

Inflammation and pain management post-surgery are crucial for recovery (12). Elevated levels of inflammatory cytokines and pain mediators could prolong recovery and negatively impact the life quality of patients. Techniques that minimize tissue disruption and reduce the inflammatory response are therefore highly desirable. Our study combined multiple assessment parameters to offer a holistic evaluation of these techniques. We examine surgical impact and early postoperative recovery, including incision length, intraoperative blood loss, and time to weight-bearing. The study also evaluates the inflammatory response and pain levels by measuring specific inflammatory cytokines [interleukin (IL)-6, IL-1β, IL-8] and pain mediators [Prostaglandin E2 (PGE2), Substance P]. Furthermore, we analyze bone metabolism through bone formation and resorption markers. By examining these diverse parameters, we seek to provide a nuanced understanding of the relative benefits and drawbacks of each technique. Through this study, we aim to contribute to the evolving body of knowledge in pediatric orthopedics, potentially leading to improved treatment strategies, reduced recovery times, and better long-term outcomes for adolescent patients with femoral shaft fractures.

## Methods

### Patients

The study was approved by Liaocheng People's Hospital. Written consent was waived since this is a retrospective study. This retrospective clinical study included 124 patients with adolescent femoral shaft fractures treated in our department. The patients were divided into two groups: 62 treated with AIN and 62 treated with locking plate fixation. The inclusion criteria were: age 11–16 years, body weight 49–59 kg, high-energy trauma as the cause of injury, unilateral femoral shaft fracture, postoperative follow-up of at least 1 year with complete clinical and imaging data from injury to bony union, and surgery performed within 7 days of injury. The exclusion criteria were: pathological fractures, open fractures, concurrent fractures in other parts of the limbs, associated vascular or nerve injuries in the lower limbs, and underlying conditions such as osteogenesis imperfecta or neuromuscular diseases. To minimize selection bias, our study utilized propensity score matching for patient selection. The matching criteria included age, body mass index (BMI), injury cause, and AO classification of fracture, with a caliper value of 0.05.

### Surgical techniques

#### Adolescent intramedullary nailing (AIN)

Patients in the AIN group were treated using the Expert Adolescent Lateral Femoral Nail System provided by Synthes. Following general anesthesia or combined spinal-epidural anesthesia, patients were positioned supine on an orthopedic surgical table. After routine sterilization and draping, a longitudinal incision was made at the proximal end of the greater trochanter. Subcutaneous fat and fascia overlying the gluteus maximus were incised, and the gluteus medius muscle fibers were separated to expose the greater trochanter. Using a 20 mm distal to the lesser trochanter as a reference point and a 12° lateral deviation at the greater trochanter as the entry point, a guidewire was inserted. The medullary canal was progressively enlarged with a flexible reamer, and the intramedullary nail was inserted. Under C-arm fluoroscopic guidance, the fracture was reduced satisfactorily, and locking screws and an end cap were inserted using a targeting arm. The surgical field was irrigated, and the wound was closed in layers. Postoperative management included routine anti-infection treatment and early quadriceps muscle contraction training without weight-bearing, progressing to gradual weight-bearing at 6–8 weeks.

#### Locking plate fixation

In the locking plate group, a 6–8 cm longitudinal incision centered over the fracture site was made. The fascia lata was incised, exposing the vastus lateralis muscle. The lateral intermuscular septum was identified, and dissection continued along the plane between the vastus lateralis and the septum to expose the femoral shaft. After clearing hematoma and soft tissue from the fracture site, reduction was achieved with the aid of a reduction device. A locking plate was then implanted, ensuring the distal end was at least 20 mm from the distal femoral epiphysis. Screws were inserted through small incisions at both ends of the plate. Satisfactory reduction was confirmed under C-arm fluoroscopy, the surgical field was irrigated, and the wound was closed in layers. Postoperative care included dressing changes every 2 days. From the second postoperative day, patients began non-weight-bearing functional exercises, including early hip and knee joint movements and later non-weight-bearing walking exercises.

### Measurement and analysis

#### Proximal femoral geometry

Bilateral proximal femoral geometric parameters were measured on anteroposterior radiographs at the final follow-up using imaging software. Two experienced residents performed the measurements, focusing on the maximum diameters of the femoral neck and femoral head.

#### Non-arthritic hip score (NAHS)

Hip function was assessed using the Non-Arthritic Hip Score (NAHS), which is suitable for young patients without hip arthritis. A score of 100 indicates normal hip function, with lower scores indicating worse hip function.

#### Collection of venous blood

Fasting venous blood samples were collected from the pediatric patients before surgery and 1 week postoperatively. Serum was separated and stored at −80°C for further analysis.

#### Inflammatory markers

Postoperative inflammation and pain were assessed by analyzing serum levels of inflammatory cytokines and pain mediators 1 week postoperatively. The extent of the inflammatory response was correlated with the degree of surgical trauma. The concentrations of inflammatory cytokines and pain mediators in serum were measured using enzyme-linked immunosorbent assay (ELISA) kits according to the manufacturers' instructions. The following kits were used: Human IL-6 ELISA Kit (Cat# EH0201), Human IL-1β ELISA Kit (Cat# EH0185), Human IL-8 ELISA Kit (Cat# EH0205), Human PGE2 ELISA Kit (Cat# EH4233)—all purchased from Wuhan Fine Biotechnology Co., Ltd (Wuhan, China). Pain mediator P was detected using an ELISA kit (Cat# E-EL-0067) purchased from Elabscience Biotechnology Co., Ltd (Wuhan, China).

#### Bone metabolism indicators

Bone formation and resorption were evaluated 1 month postoperatively by measuring serum levels of bone alkaline phosphatase (BALP), bone gamma-carboxyglutamate protein (BGP), and beta-crosslaps (β-CTX) using ELISA kits according to the manufacturers' instructions. The following kits were used: Human BALP ELISA Kit (Cat# EH2691), Human OC/BGP ELISA Kit (Cat# EH3468), Human β-CTx ELISA Kit (Cat# EH3989). All kits were purchased from Wuhan Fine Biotech Co., Ltd. These indicators were analyzed to understand the balance of bone metabolism and its impact on fracture healing.

Both groups underwent early functional exercise, and all patients completed follow-up ranging from 19 to 48 months. At the final follow-up, all fractures had achieved bony union without significant limitations in hip or knee joint mobility. Radiographic evaluations revealed no complications such as femoral head necrosis or hip valgus deformity.

### Statistical analysis

Data were expressed as *n* (percentage, %) or mean ± standard deviation (SD). The normality of the data before analysis was examined using Anderson–Darling test. The comparisons of data between the two groups were done by unpaired *t* test with Welch's correction, Chi-square test or Fisher's exact test. The significant statistical difference was determined when *p* values were less than 0.05.

## Results

### The demographic and clinical characteristics of the participants

The demographic and clinical characteristics of adolescents with femoral shaft fractures treated with either AIN or LP were summarized in Table 1. Propensity score matching was employed to minimize selection bias, using age, BMI, injury cause, and AO classification of fracture as matching criteria, with a caliper value of 0.05. The process of the subject selection was shown in Supplementary Figure S1. The comparison revealed no significant differences between the two groups in terms of age (AIN: 13.75 ± 1.24 years, LP: 13.69 ± 1.38 years, *p* = 0.582), BMI (AIN: 23.35 ± 0.49 kg/m^2^, LP: 23.16 ± 0.57 kg/m^2^, *p* = 0.184), gender distribution (boys: AIN 61.3%, LP 53.2%; girls: AIN 38.7%, LP 46.8%; *p* = 0.468), injury cause (accident: AIN 32.3%, LP 40.3%; fall injury: AIN 46.8%, LP 45.2%; sports injury: AIN 20.9%, LP 14.5%; *p* = 0.522), and AO classification of the fracture (A: AIN 61.3%, LP 58.2%; B: AIN 24.2%, LP 20.9%; C: AIN 14.5%, LP 20.9%; *p* = 0.630).

**Table 1:** Demographic and clinical characteristics of femoral shaft fractures adolescent received the treatment of adolescent intramedullary nail (AIN) or locking plate (LP).

Characteristics	AIN (*n* = 62)	LP (*n* = 62)	*p* value
Age (years)	13.8 ± 1.2	13.7 ± 1.4	0.582
BMI (kg/m^2^)	23.4 ± 0.5	23.2 ± 0.6	0.184
Gender			
Boy	38 (61.3%)	33 (53.2%)	0.468
Girl	24 (38.7%)	29 (46.8%)	
Injury cause			
Accident	20 (32.3%)	25 (40.3%)	0.522
Fall injury	29 (46.8%)	28 (45.2%)	
Sports injury	13 (20.9%)	9 (14.5%)	
AO classification of fracture			
A	38 (61.3%)	36 (58.2%)	0.630
B	15 (24.2%)	13 (20.9%)	
C	9 (14.5%)	13 (20.9%)	

Values were expressed as *n* (percentage, %) or mean ± SD. The comparisons of data between the two groups were done by Mann–Whitney test. Chi-square test or Fisher's exact test was used for assessing distribution of observations or phenomena between different groups.

### Surgical impact and early postoperative recovery

**Figure 1:**
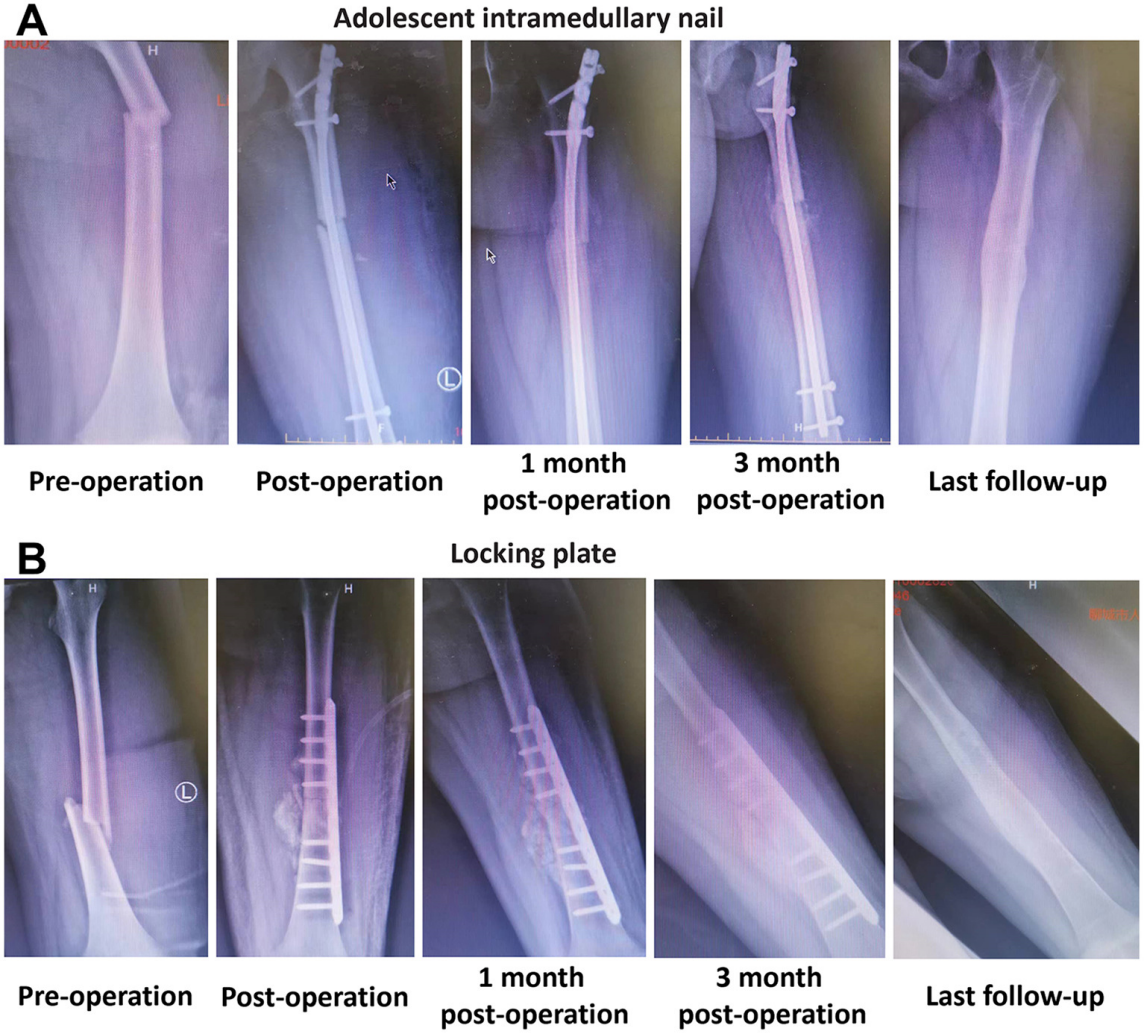
Typical cases of femoral shaft fractures adolescent received the treatment of adolescent intramedullary nail (AIN) or locking plate (LP). **(A)** X-ray images of a 13 years old boy with left femoral shaft fracture received the treatment of AIN at different times. **(B)** X-ray images of a 14 years old boy with left femoral shaft fracture received the treatment of LP at different times.

Figure 1 illustrated the progression of fracture healing and the stability of fixation methods in adolescents treated with either an AIN or a LP over time. The typical x-ray images of a 13-year-old boy with a left femoral shaft fracture treated with AIN are shown at different stages: pre-operation, post-operation, 1-month post-operation, 3 months post-operation, and at the last follow-up (Figure 1A). The corresponding x-ray images for a 14-year-old boy with a left femoral shaft fracture treated with LP are also shown at these stages (Figure 1B). The images in both panels highlight the sequential healing process and the effectiveness of the respective treatment methods in maintaining fracture alignment and promoting bone healing.

**Figure 2:**
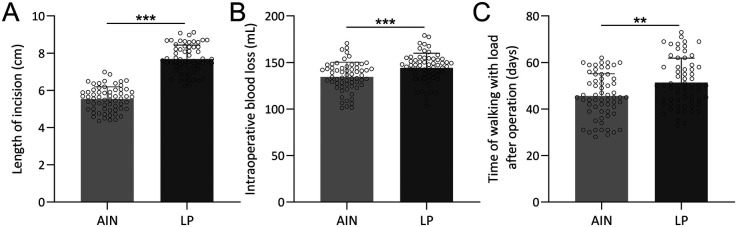
Comparisons of length of incision **(A)**, intraoperative blood loss **(B)** and time of walking with load after operation **(C)** between femoral shaft fractures adolescent received the treatment of adolescent intramedullary nail (AIN) or locking plate (LP). *n* = 62 for each group. Data were shown with mean ± SD. **p < 0.01, ***p < 0.001 from Unpaired *t* test with Welch's correction.

The comparisons between the AIN and LP groups revealed significant differences in several surgical and early postoperative parameters (Figure 2). The incision length was significantly shorter in the AIN group (*p* < 0.001), with a mean incision length of 6.8 cm compared to 8.5 cm in the LP group (Figure 2A). Intraoperative blood loss was also notably lower in the AIN group, averaging 120 ml compared to 170 ml in the LP group (*p* < 0.001, Figure 2B). Additionally, the time to walking with load post-operation was significantly shorter for the AIN group, with patients beginning weight-bearing activities at an average of 34 days post-surgery compared to 47 days in the LP group (*p* < 0.01, Figure 2C). These findings indicate that the AIN procedure is less invasive and facilitates earlier mobilization compared to the LP method.

### Proximal femoral geometry and hip function

**Figure 3:**
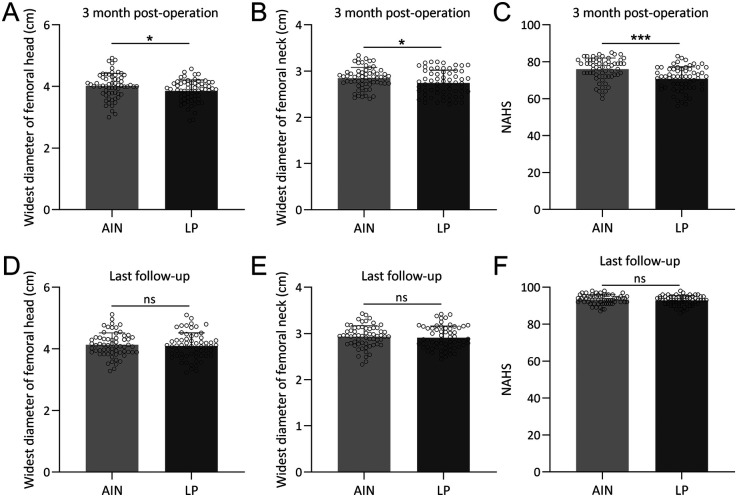
Comparisons of widest diameter of femoral head **(A)**, widest diameter of femoral head **(B)**, NAHS **(C)** between the two groups at the time of 3 months post-operation and also at the time of last follow-up **(D–F)**. *n* = 62 for each group. Data were shown with mean ± SD. *p < 0.05, **p < 0.01, ***p < 0.001 from Unpaired *t* test with Welch's correction.

At 3 months post-operation, significant differences were observed in the widest diameter of the femoral head and the NAHS between the two groups (Figure 3). The AIN group exhibited a larger widest diameter of the femoral head and neck (*p* < 0.05, Figures 3A,B) and higher NAHS (*p* < 0.001, Figure 3C), suggesting better early postoperative hip function and geometry. Specifically, the mean NAHS was 87 in the AIN group vs. 75 in the LP group. However, by the last follow-up, these differences were no longer significant, with both groups showing comparable outcomes in the widest diameter of the femoral neck and head, as well as in the NAHS (Figures 3D–F). This convergence indicates that while both treatment methods ultimately lead to satisfactory long-term recovery, the AIN group achieves faster initial improvements in hip function and femoral geometry.

### Intramedullary nailing reduces inflammation and pain in adolescent femoral fractures

We next compared the levels of serum inflammatory cytokines and pain mediators between the AIN and LP groups 1-week post-operation. To establish baseline comparability, the serum levels of these inflammatory markers and pain mediators pre-operation were assessed and the data confirm that there were no significant differences between the groups before surgery (Supplementary Figures S2A–E).

**Figure 4:**
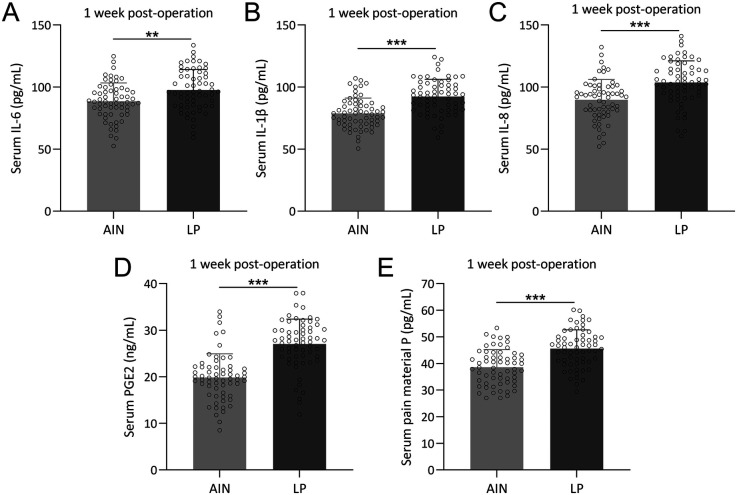
Comparisons of serum IL-6 **(A)**, IL-1β **(B)**, IL-8 **(C)**, PGE2 **(D)** and pain material P **(E)** between the two groups at the time of 1 week post-operation. *n* = 62 for each group. Data were shown with mean ± SD. **p < 0.01, ***p < 0.001 from Unpaired *t* test with Welch's correction.

However, there were significant differences in the levels of inflammatory markers and pain mediators between the two groups. Specifically, the LP group had higher serum concentrations of IL-6 (Figure 4A), IL-1β (Figure 4B), IL-8 (Figure 4C), PGE2 (Figure 4D), and pain mediator P (Figure 4E) compared to the AIN group. These results indicate that the AIN method is associated with a lower inflammatory response and reduced pain levels 1-week after surgery, suggesting it is less traumatic and more effective in minimizing postoperative pain.

### Enhanced bone formation and reduced resorption in AIN

**Figure 5:**
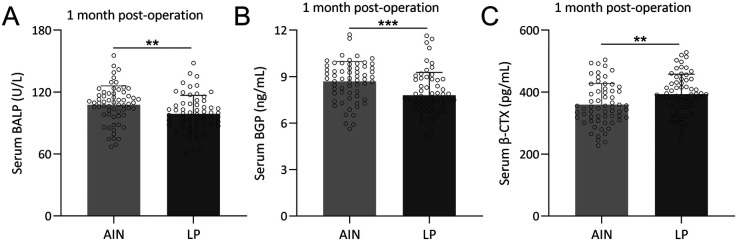
Comparisons of serum BALP **(A)**, BGP **(B)** and β-CTX **(C)** between the two groups at the time of 1-month post-operation. *n* = 62 for each group. Data were shown with mean ± SD. **p < 0.01, ***p < 0.001 from Unpaired *t* test with Welch's correction.

To compare the levels of bone formation and resorption markers between the AIN and LP groups 1-month post-operation, we assessed the bone metabolism indicators between the two groups. There were no significant differences in serum levels of BALP, BGP, and β-CTX between the groups before surgery (Supplementary Figures S3A–C). Interestingly, 1-month after operation, the AIN group showed higher levels of bone formation markers BALP (Figure 5A) and BGP (Figure 5B), and lower levels of the bone resorption marker β-CTX (Figure 5C) compared to the LP group. These findings suggest that the AIN treatment promotes better bone formation and less bone resorption 1 month after surgery, which correlates with faster fracture healing observed in the AIN group.

## Discussion

This study compared the clinical outcomes of AIN and LP fixation in the treatment of femoral shaft fractures of adolescents. Our results highlighted significant differences in surgical impact, early postoperative recovery, inflammatory response, pain levels, and bone metabolism markers, providing valuable insights into the relative efficacy and safety of these treatment modalities.

### Surgical impact and early postoperative recovery

Our findings indicated that AIN was less invasive than LP, as evidenced by significantly shorter incision lengths and lower intraoperative blood loss. Specifically, the mean incision length in the AIN group was 6.8 cm, compared to 8.5 cm in the LP group, and the average blood loss was 120 vs. 170 ml, respectively. These differences suggest that the AIN procedure minimizes tissue damage and bleeding, leading to a more favorable surgical profile. Additionally, patients in the AIN group began weight-bearing activities on average 34 days post-surgery, significantly earlier than the 47 days observed in the LP group. Early mobilization is crucial for reducing the risk of postoperative complications such as deep vein thrombosis and muscle atrophy, and it contributes to overall better recovery outcomes (13–15). Similar trends were reported that minimally invasive approaches in femoral fracture repair were associated with reduced soft tissue disruption and faster rehabilitation (16).

### Proximal femoral geometry and hip function

The AIN group exhibited superior early postoperative hip function and proximal femoral geometry compared to the LP group at 3 months post-operation. The widest diameters of the femoral head and neck were significantly larger in the AIN group, and the NAHS was higher, with a mean score of 87 compared to 75 in the LP group. These metrics suggest that AIN better preserves the anatomical integrity of the femur and supports hip function during the initial recovery phase. However, by the last follow-up, the differences in proximal femoral geometry and NAHS between the two groups were no longer significant, indicating that both treatment methods achieve similar long-term recovery outcomes. This convergence implies that while AIN offers faster initial improvements, both methods are effective in restoring function and anatomy over time.

A previous study that retrospectively analyzed the functional outcomes of LP vs. AIN for extra-articular distal femoral fractures noted a faster, albeit not statistically significant, union rate for AIN compared to LP (17). Our results similarly indicated that AIN generally leads to quicker fracture healing, supporting its advantage in facilitating faster recovery. Additionally, a meta-analysis of 20 studies involving 1,384 patients with proximal humeral fractures found that intramedullary nails were superior to LP in terms of shorter incision length, less peri-operative bleeding, reduced operation time, and faster fracture healing (18). These findings are consistent with our observations. Another study reported that AIN resulted in significantly lower shoulder pain and higher median Constant and ASES scores compared to LP, with fewer complications and revision surgeries (19). Collectively, these studies reinforce that while both AIN and LP are effective in achieving long-term clinical outcomes, AIN offers distinct advantages in the early postoperative period and in reducing complications.

Furthermore, similar patterns have been observed in femoral neck fracture treatments, where minimally invasive intramedullary fixation techniques were associated with improved short-term hip function and preservation of proximal femoral alignment (20). Moreover, biomechanical analyses have demonstrated that intramedullary implants distribute load more efficiently along the femoral axis compared to lateral plates, which may explain the superior early outcomes observed in our study. For instance, a biomechanical comparison using synthetic bone models found that proximal femoral intramedullary nails sustained approximately 1.78-fold greater axial load before failure than locking proximal anatomic femoral plates, highlighting their superior load-sharing capacity under axial compression (21). Additionally, finite element analysis studies have shown that intramedullary nails bear higher von Mises stresses and transmit loads centrally through the bone's mechanical axis, reducing stress concentration in the implant—a biomechanical advantage that correlates with improved early fracture stabilization (22).

### Intramedullary nailing reduces inflammation and pain

Intramedullary nailing can trigger a “second hit” effect on a patient physiology, which includes several inflammatory and physiological responses (23–25). This second hit is caused by increased pressures within the intramedullary canal, intravasation of fat particles, activation of coagulation, marrow embolization to the lungs or brain, and overheating of the endosteum. Femoral reaming is associated with increased blood loss from the fractured extremity.

Various biomarkers have been studied to understand the cumulative effects of these factors during AIN (26). Key biomarkers such as IL-6, IL-8, IL-10, and TNF-α have been identified. IL-6, in particular, is a significant marker of the early post-traumatic immune response and is elevated in both reamed and unreamed nailing. Studies have shown that the type of surgical procedure, initial trauma severity, specific injury combinations, and timing of surgical intervention are critical factors influencing the inflammatory response (27). The period between 48 h and the 5th day post-trauma is identified as suboptimal for major surgeries due to the heightened risk of systemic inflammatory response syndromeand complications (26, 28). Effective resuscitation and careful timing of surgical interventions are crucial to mitigating the second hit's impact. Here, we found that 1-week after operation, the LP group had significantly higher serum levels of inflammatory cytokines IL-6, IL-1β, and IL-8, as well as pain mediators PGE2 and pain mediator P, compared to the AIN group. These elevated levels indicate a higher inflammatory response and greater pain in the LP group, suggesting that the AIN method is less traumatic and more effective in minimizing postoperative pain. Reduced inflammation and pain are associated with faster recovery and improved patient comfort, underscoring the advantages of AIN in postoperative management (29).

### Enhanced bone formation and reduced resorption in AIN

BALP and BGP were used as bone formation markers, and β-CTX was employed as a bone resorption marker. BALP promotes bone mineralization (30), while BGP regulates bone matrix crystallization and reflects recent osteoblast activity (31, 32). β-CTX, released during increased osteoclast activity, is the gold standard for assessing bone resorption (33). Bone metabolism markers provide insight into the biological processes underlying fracture healing. One-month post-operation, the AIN group demonstrated higher levels of bone formation markers BALP and BGP and lower levels of the bone resorption marker β-CTX compared to the LP group. These findings suggest that AIN promotes better bone formation and less bone resorption, correlating with faster and more robust fracture healing. Enhanced bone formation is crucial for the stabilization and consolidation of fractures, while reduced bone resorption minimizes the risk of delayed union or nonunion. The absence of significant differences in these markers pre-operation further supports the postoperative benefits observed with AIN.

Our study comparing AIN and LP fixation for adolescent femoral shaft fractures revealed significant advantages of AIN in terms of surgical impact, early recovery, and bone metabolism. These findings align with broader research in pediatric orthopedics. Chin See et al. demonstrated the efficacy of rigid intramedullary nailing in children with metabolic bone diseases, supporting its application beyond typical cases (34). Another study explored bioabsorbable intramedullary nails for pediatric forearm fractures, highlighting potential benefits in eliminating secondary surgeries but also noting technique-dependent complications (35). For overweight pediatric patients with narrow medullary canals, Tang et al. proposed combining elastic stable intramedullary nails with temporary external fixators as an alternative to locking compression plates, showing improved outcomes in operation time, blood loss, and union rates (36). Collectively, these studies, including our own, underscore the importance of tailoring fixation methods to patient-specific factors such as age, weight, bone quality, and fracture characteristics. While AIN demonstrates clear advantages in our adolescent cohort, the evolving landscape of pediatric orthopedic fixation techniques suggests a nuanced approach is necessary. Future research should focus on refining selection criteria for various fixation methods, optimizing techniques to minimize complications, and conducting long-term follow-up studies to ensure sustained positive outcomes across diverse patient populations.

## Conclusion

In conclusion, AIN offers significant advantages over LP in the treatment of adolescent femoral shaft fractures, including reduced surgical trauma, faster early recovery, lower inflammatory response, less postoperative pain, and enhanced bone healing. These benefits make AIN a more favorable option for managing these types of fractures, particularly in the early postoperative period. While both treatment methods ultimately achieve satisfactory long-term outcomes, the faster initial recovery associated with AIN provides a compelling case for its use in clinical practice.

## Data Availability

The raw data supporting the conclusions of this article will be made available by the authors, without undue reservation.
