# Supplementary material for: Comparative analysis of adolescent intramedullary nailing and locking plate fixation for femoral shaft fractures
Source: Front Surg. 2025 Jul 17;12:1614146. doi: 10.3389/fsurg.2025.1614146 (PMC12310659; doi:10.3389/fsurg.2025.1614146)
Supplement: Supplementary file 1 [file Table1.docx]

**Supplementary materials**

**
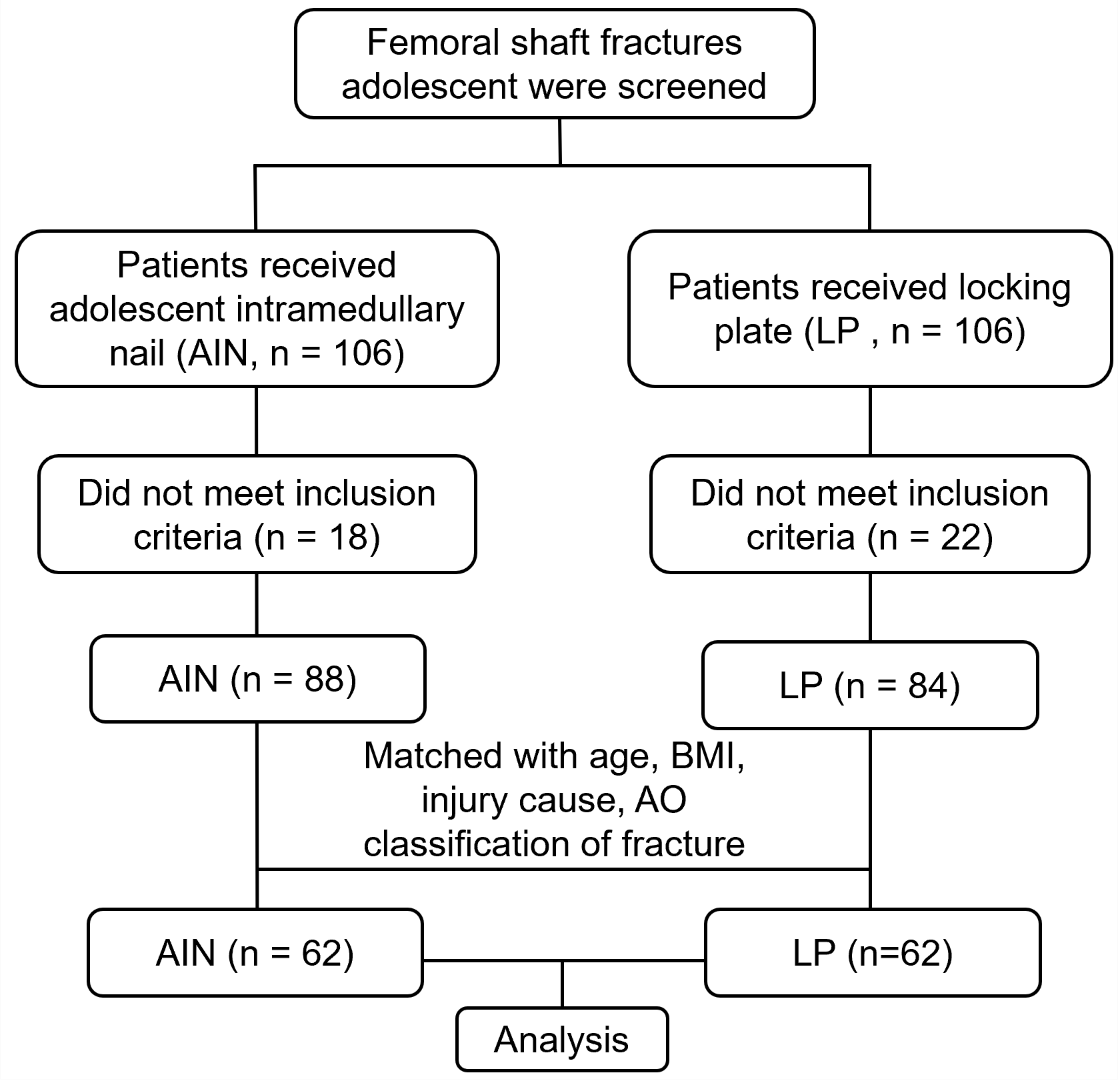
**

**Figure S1**. Propensity score matching process of the subject selection.

**
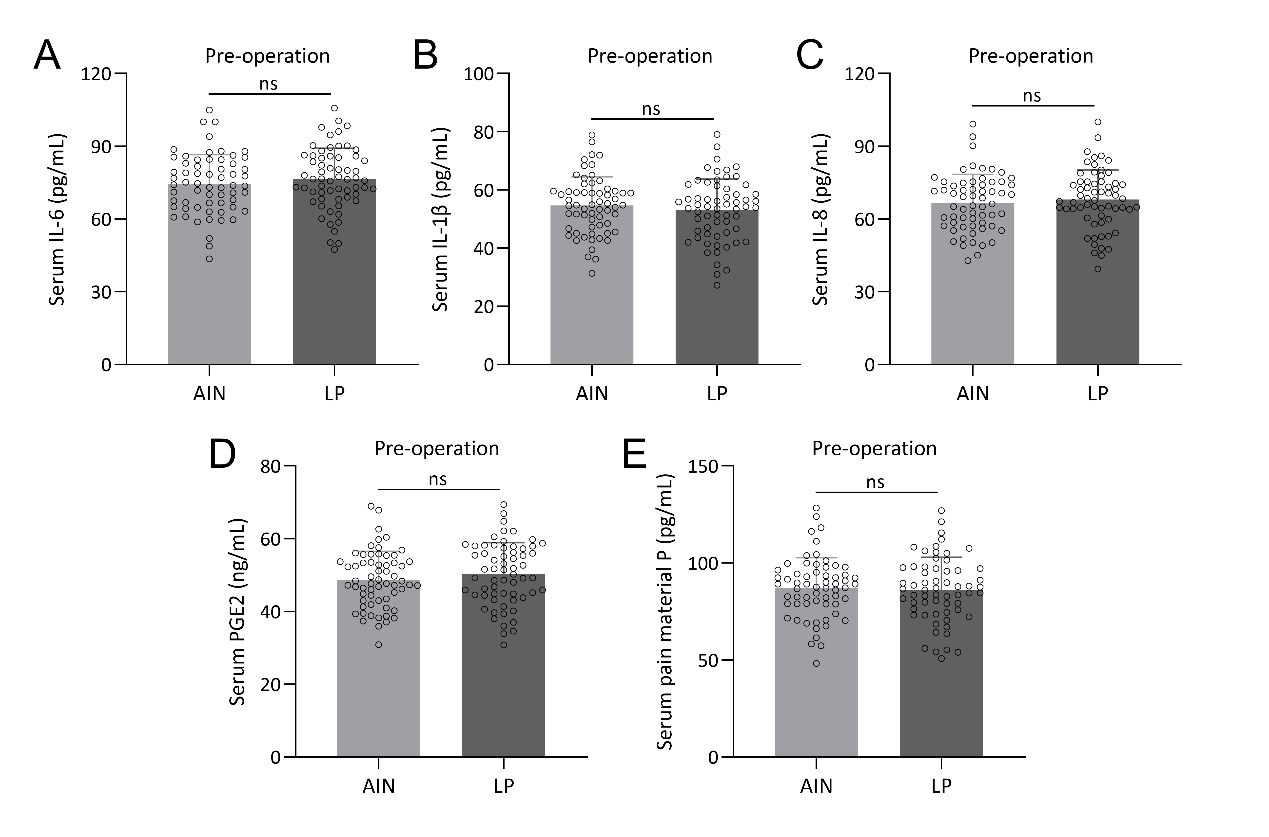
**

**Figure S2.** Comparisons of serum IL-6 (A), IL-1β (B), IL-8 (C), PGE2 (D) and pain material P (E) between the two groups at the time of pre-operation. n = 62 for each group. Data were shown with mean ± SD. ns means no significance from Unpaired t test with Welch's correction.


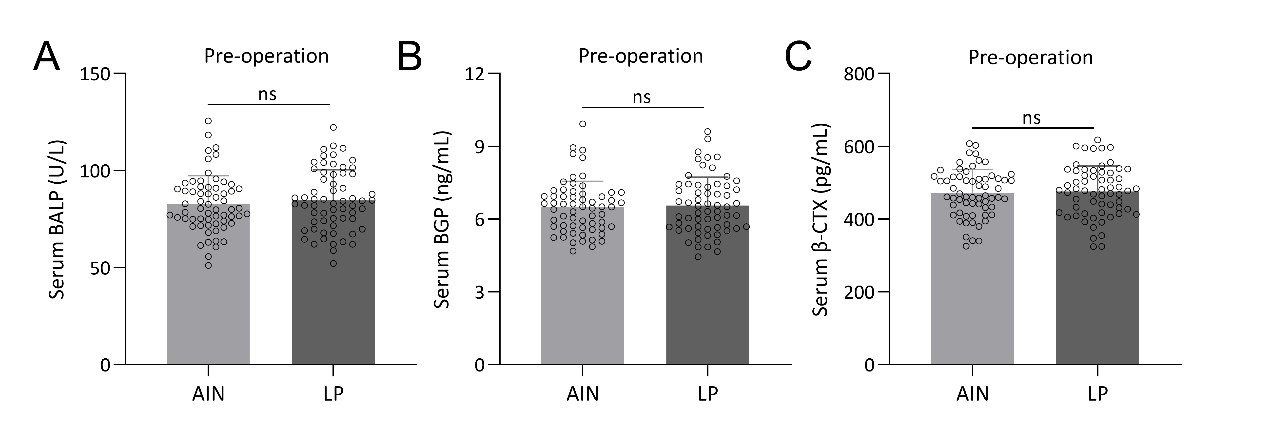


**Figure S3.** Comparisons of serum BALP (A), BGP (B) and β-CTX (C) between the two groups at the time of pre-operation. n = 62 for each group. Data were shown with mean ± SD. ns means no significance from Unpaired t test with Welch's correction.
